# Ectopic thoracic thyroid removed by uniportal VATS approach. A case report

**DOI:** 10.1016/j.ijscr.2019.07.027

**Published:** 2019-07-19

**Authors:** F. Carannante, L. Frasca, M. Depalma, F. Longo, P. Crucitti

**Affiliations:** Department of Thoracic Surgery, Università Campus Bio-Medico, Via Alvaro del Portillo 21, 00128 Rome, Italy

**Keywords:** Thoracic surgery, Uniportal VATS, Ectopic thyroid gland, Minimally invasive surgery

## Abstract

**Introduction:**

We speak of “Ectopic thyroid gland” when a piece of thyroid tissue is placed at a certain distance from the second to fourth tracheal cartilages.

**Presentation of case:**

This case report focuses on a case of a hyperplastic cystic nodule of ectopic thyroid in a 30-year-old woman treated with uniportal video-assisted thoracic surgery (VATS). The patient, was admitted to Emergency Unit for abdominal pain and vomit, underwent a CT which highlighted a mass of significant dimension on the right side of the mediastinum, in contact with close structures. The lesion has been removed with an innovative mini-invasive technique, which is characterized, differently from traditional surgical approaches, by reduced loss of blood and time of hospitalization and, in addiction to that, an aesthetic small-dimensioned scar.

**Discussion:**

VATS technique is minimally invasive and, moreover, it is characterized by a shorter recovery period, a minor blood loss and a shorter hospital stay. VATS has been advocated since 2010 for pulmonary resections, but today it is also performed for mediastinal intervention and a series of reports have demonstrated that it is feasible and safe.

**Conclusion:**

The report aims at pointing out the possibility of undergoing this kind of surgery not only in case of pulmonary mass (on which the previous literature has focused) but also when mediastinum lesions occur, as in our case.

## Highlights

• VATS technique is minimally invasive and, moreover, it is characterized by a shorter recovery period, a minor blood loss and a shorter hospital stay.
• We speak of “Ectopic thyroid gland” when a piece of thyroid tissue is placed at a certain distance from the second to fourth tracheal cartilages.
• This case report aims at describing the successful extraction of a massive piece of ectopic thyroid from a young woman’s body, thanks to uniportal VATS.

## 1 Introduction

From an embryological point of view, the thyroid derives from the epithelial proliferation of the pharyngeal floor. Afterwards the gland migrates ahead of the pharyngeal gut and assumes a bilobed aspect. During this transition, the thyroid adheres to the tongue by means of a thyroglossal duct, which thereafter disappears [1].

We speak of “Ectopic thyroid gland” when a piece of thyroid tissue is placed at a certain distance from the second to fourth tracheal cartilages. It is considered to be a rare malformation which occurs in 1 case out of 100,000–300,000 people [1]. Since during the embryogenic development the gland transmigrates from the floor of the primitive foregut to the neck, it is more common to find this kind of lesion in the neck and thorax [2]. Other reported locations are trachea, heart, lung, duodenum, adrenal gland, gall bladder, porta hepatis, esophagus, parotid salivary gland [3,4].

It is important to differentiate the mediastinum thyroid from germ cell tumors, neurogenic tumors, lymphomas, mesenchymal and thymic tumors [5].

In addition to this, cases of ectopic thyroid cancers are considered to be rare, as well as the real mediastinal thyroid cancer (one reported case in 2006) [6].

Ectopic mediastinal thyroid is normally asymptomatic and does not compromise any thyroidal function. However, it is possible to observe symptoms like chest pain, cough, dyspnea and those deriving from compression of V. Cava [7].

In order to distinguish ectopic thyroid from secondary goiters, we should consider the following criteria: the presence of an independent blood supply by intrathoracic vessels instead of cervical ones, the absence of abnormalities of cervical thyroid gland, no history of malignancy [8].

We report a particular case of a 30-year-old woman with an expansive right paratracheal lesion who underwent uniportal video-assisted thoracic surgery (VATS) to remove the mass.

This work has been reported in line with the SCARE criteria [10].

## 2 Case presentation

**Fig. 1:**
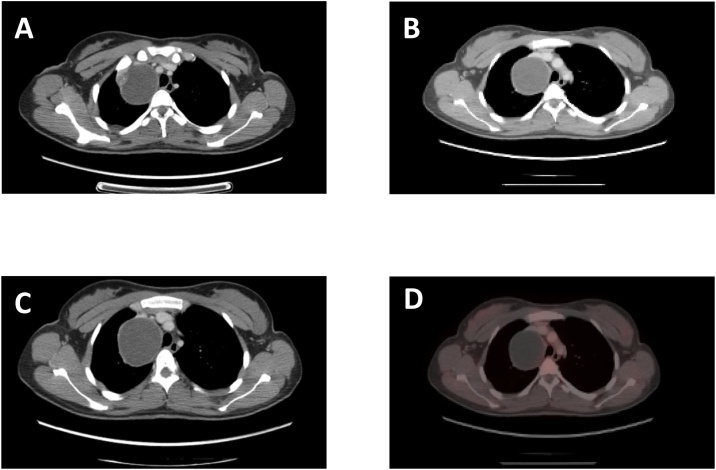
Chest CT in axial views of the heterogeneous right side anterior lateral mass (A, B,C); Chest PET of the mass (D).

A 30-year-old woman was admitted to Emergency Department for abdominal pain and vomit. She was a student, not a smoker, from southern Italy. A CT scan showed an expansive roundish capsulated cystic lesion (58 mm × 71 mm) with solid projections. The mass, located on the right side of the mediastinum, impinged and dislodged anteriorly the right Superior V. Cava and displaced oppositely the trachea (Fig. 1A–C). The lesion did not catch at CT/PET (Fig. 1D). At first, it was thought to be a teratoma.

We decided to remove the mass through a Uniportal Video-assisted Thoracoscopic Surgery, performed by Professor Crucitti.

We started the intervention performing a mini-thoracotomy at the IV intercostal space along the right medium axillary line. Once the access to the pleural cavity had been gained, an Alexis size S depressor was positioned. The mass adhered to the right Superior V. Cava and Azygos vein, which appeared narrowed and not completely cleavable from neoplasia. In addition to that, the lesion adhered to the Phrenic nerve and the Vagal nerve. The neo-formation was isolated from the structures mentioned above and from the tracheal surface. The Azygos vein was isolated and sectioned upstream of the lesion and of the caval confluence, using an echelon flex stapler. The extraction was made through the protection of an endobag, but the mass was larger in its dimension than the incision made, so we have sucked up part of the liquid from the mass (Fig. 5). No intraoperative complications occurred. A pleural drainage was performed (Fig. 3B).

No post-operative complications occurred. The pleural drainage was removed on the first post-operative day and on that same day the patient left the Hospital.

**Fig. 2:**
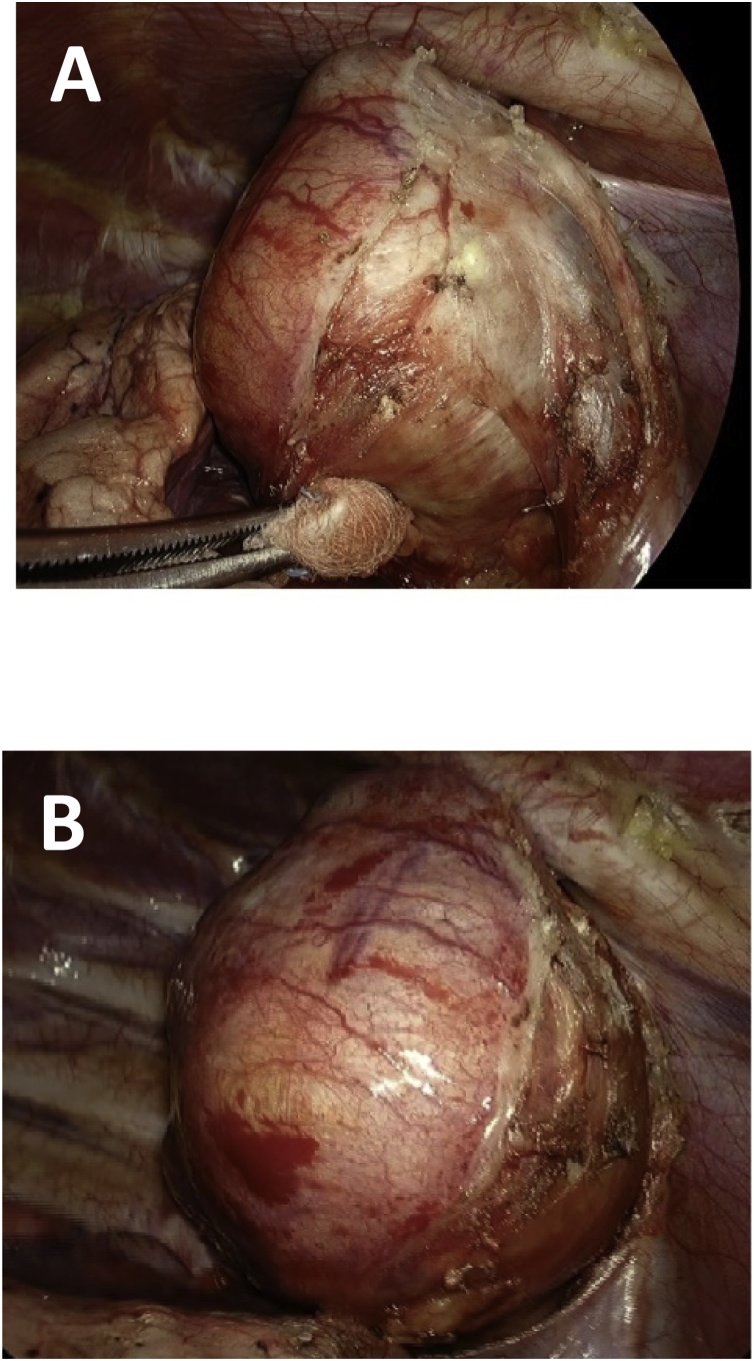
Intra-operative images (A, B).

Subsequent anatomo-histological exams identified it as a hyperplastic colloidal nodule belonging to the thyroid gland with haemorrhagic and cystic aspect (Fig. 2). According to the analysis, its proper dimension was the following: 65 mm × 60 mm × 28 mm. Weight: 98 g. (Fig. 4) (Fig 6)

30 days after the surgical procedure the patient underwent a follow-up thoracic CT without mdc. The woman is currently in good clinical general conditions, without any kind of distress surgery-related.

## 3 Discussion

Nonetheless, the ectopic thyroid was non-functional, its considerable dimension and the compression exerted on the adjacent structures (vessels, nerves, organs) justified the surgical removal. The major techniques for performing anterior mediastinal tumour resection are midline exploratory sternotomy, anterior lateral thoracotomy and VATS.

**Fig. 3:**
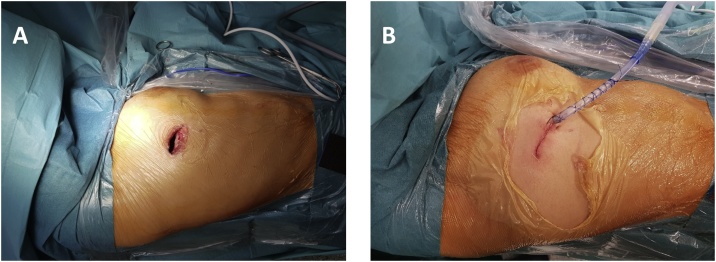
Wound before (A) and after suture (B). Picture B shows the placement of a transthoracic drainage.

VATS technique is minimally invasive and, moreover, it is characterized by a shorter recovery period, a minor blood loss and a shorter hospital stay. In addition, the result of this type of surgery is not less impressive than open surgery [9]. VATS and robot-assisted techniques have become popular because not only they allow to remove the hole mass, but also because they meet the cosmetic desires of the patients, such as a scar as reduced in its size as possible (Fig. 3). Their only limit is that, nowadays, they seem to be unsuitable for tumours that are bigger than 10 cm. VATS has been advocated since 2010 for pulmonary resections, but today it is also performed for mediastinal intervention and a series of reports have demonstrated that it is feasible and safe [9]. In spite of this, literature is still lacking in cases of ectopic thyroid removal by VATS. Surgeons, who have always been struggling with themselves to be technically innovative and safe at the same time, have found this technique useful to remove masses of significant dimensions with no major bleeding during surgery. According to Diego Gonzalez-Rivas, giant tumours may impede an accurate use of instruments and also a proper operation view during open surgery, which might cause bleeding and other complications [9].

**Fig. 4:**
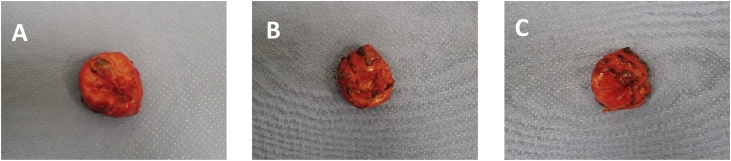
Surgical specimen of the lesion (A, B, C).

**Fig. 5:**
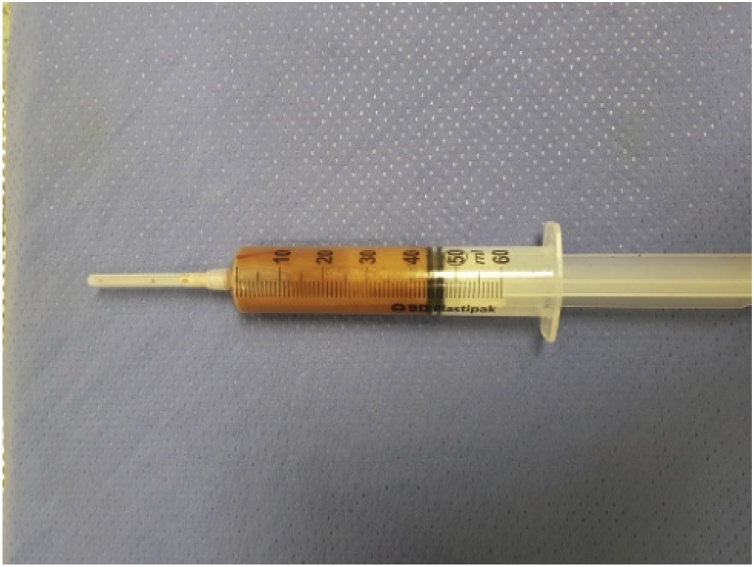
Fluid removed from the mass before the extraction.

**Fig. 6:**
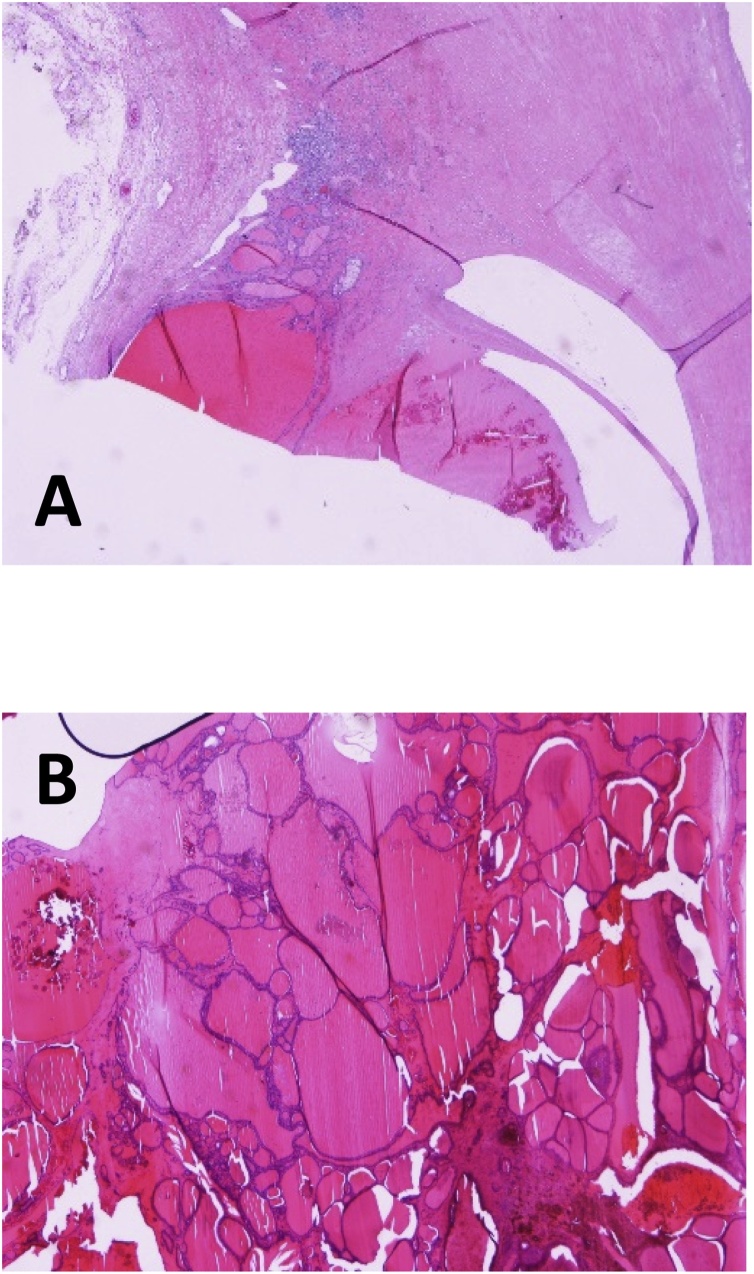
Microphotograph of ectopic thyroid fixed with Hematoxylin and eosin (A, B).

## 4 Conclusions

This case report aims at describing the successful extraction of a massive piece of ectopic thyroid from a young woman’s body, thanks to uniportal VATS. This technique has a number of advantages, such as the limited period of hospital stay (2 days in our case) and better cosmetic results, differently from the traditional techniques (sternotomy, thoracotomy, triportal thoracoscopy).

In conclusion, we believe that, if performed by experienced surgeons, this mini-invasive technique should be preferred.

## Sources of funding

The Authors disclose no sources of funding for research.

## Ethical approval

This is a case report. It’s exempt from ethical approval.

## Consent

Written informed consent was obtained from the patient for publication of this case report and accompanying images. A copy of the written consent is available for review by the Editor-in-Chief of this journal on request.

## Author contribution

F. Carannante: study design, data collections, data analysis, and writing.

L. Frasca: data collections, data analysis.

M. Depalma: study design, data collections, data analysis, and writing.

F. Longo: study design.

P. Crucitti: reviewer.

## Registration of research studies

This study is not a first in man study and the registration in a publicly accessible database is not required.

## Guarantor

Dr. P. Crucitti.

## Provenance and peer review

Not commissioned, externally peer-reviewed.

## Declaration of Competing Interest

The authors disclose no conflicts.
